# The Irish Medical Association

**Published:** 1917-04-28

**Authors:** 


### THE EDITOR'S LETTER-BOX.

### THE IRISH MEDICAL ASSOCIATION.

### Some Extraordinary Resolutions.

*To the Editor of* The Hospital.

**[We have received from the Secretary of the Irish Medical Association a request that publicity may be given to the following resolutions of the Council of that body.]**

The Council of the Irish Medical Association consider that it is unfair and dangerous, from a public health point of view, for the authorities to refuse sanctioning temporary appointments of men of Military age in the country, where the services of men over Military age cannot be procured.

The Council of the Irish Medical Association consider, that the Military Authorities are not consistent in their actions, as they refuse to employ Medical men for foreign service as they are over 50 years of age although active and energetic, who in some cases are well known Operating Surgeons, while they have given Military home work to many men who are, of Military age.

[The first of these two extraordinary resolutions does not specify what kind of temporary appointments the Council refers to. Those who have followed the discreditable incidents connected with medical officerships to the Guardians at Letterkenny and Omagh, or will refer to our own repeated comments on those incidents (The Hospital, January 20, 1917, p. 330; December 9, 1916, p. 197; September 23, 1916, p. 577; and September 9, 1916, p. 536) must be driven to conclude that the Council of the Irish Medical Association is in sympathy with the Nationalists and Sinn Feiners, who are doing their best to hinder recruiting in Ireland, and thereby to uphold the cause of Germany as far as in them lies. This, we submit, is a fair, and, indeed, inexorable inference from the facts.

The second resolution, which is printed exactly as received, is so ungrammatical both in wording and in punctuation, as well as so thoroughly parochial in spirit, that we can scarcely credit any body of professional men with the sending of it forth. The whole letter simply deprives the Council of the Irish Medical Association, in our opinion, of any right to be heard or considered in this matter. We sincerely trust that it speaks authoritatively for neither the I.M.A. itself nor for the Irish medical profession.—Ed. The Hospital.]

